# Crustal structure and seismic anisotropy of rift basins in Somaliland

**DOI:** 10.1038/s41598-023-44358-2

**Published:** 2023-10-14

**Authors:** Mohammed Y. Ali, Mohammad Ismaiel, Ibrahim M. Yusuf, Ayoub Kaviani

**Affiliations:** 1 Department of Earth Sciences, Khalifa University of Science and Technology, Abu Dhabi, UAE; 2 School of Geography, Earth and Environmental Sciences, University of Birmingham, Birmingham, UK; 3 Institute of Geosciences, Goethe University Frankfurt, Frankfurt, Germany

**Keywords:** Geophysics, Tectonics, Seismology

## Abstract

Rift margins provide insights into the processes governing the rupture of the continental lithosphere and the subsequence formation of sedimentary basins. The Proterozoic basement underlying Somaliland has been affected by multiple rifting; however, the crustal structure of these rifted basins remains unknown. This study utilized teleseismic receiver function analysis, Bayesian inversion, common conversion point imaging and 2D forward gravity modelling to examine the crust and upper mantle of Somaliland. The results indicate 36.8–38.2 km of crust in southern Somaliland, while the central and northern regions feature thinned crust (~ 21 km) with 5–6 km thick sediments. The joint analysis of radial and transverse components of receiver functions and shear wave splitting revealed fast axis directions trending to 50–56° in the upper mantle, indicating that azimuthal anisotropy is oriented in the regional Africa-Arabia plate motion. Such orientation may have resulted from lattice preferred orientation of olivine from the asthenospheric flow. Additionally, the fast polarization of the crust in central Somaliland is oriented at − 15°, indicating fossil deformation in the thinned crust related to the NW–SE trending Late Jurassic rift event. Further, the fast polarization for stations near the Gulf of Aden is oriented at 75–80°, suggesting crustal deformation associated with the Oligocene rift event. The crustal anisotropy at southern Somaliland revealed fast polarization oriented at − 85°, indicating a preserved far-field response of the WNW-ESE trending Late Cretaceous rift event. Overall, the study provides for the first-time insight into the rift-related extensional strain fabric in the crust and upper mantle anisotropy induced by asthenospheric flow in Somaliland.

## Introduction

Rift initiation and formation of sedimentary basins are the key steps responsible for the deformation of the Earth’s surface. Seismic anisotropy is commonly used to examine the ongoing dynamic processes and paleo-deformation history of the continental rifts e.g.,^1–3^. Many studies on continental rift systems, such as the East African Rift, show a fast azimuthal anisotropy axis parallel to the rift axis with a splitting time as large as 2.5 s^2,4–6^. The anisotropy within the lithosphere in rift zones is produced by intruded melt and spreading-parallel fabric^2,6–8^. Asthenospheric flow beneath the rift shoulders produces lattice-preferred orientation (LPO) of olivine crystals where the fast axes of the mineral aggregates line up in the divergence direction^7,9^.

The Somaliland region in northern Somalia is part of the Arabian-Nubian Shield, which is comprised of Proterozoic terranes that amalgamated during the Neoproterozoic^10^. Somaliland has been impacted by multiple rift events, which have led to the formation of sedimentary basins, thus providing a unique opportunity to study failed rift systems. The first event occurred in the Late Jurassic period, resulting in NW–SE trending grabens, which are attributed to be a result of the late-stage breakup of Gondwana and the detachment of Madagascar from Africa^11–14^. This rift led to the development of the NW–SE trending basins in northern Somalia, such as the Guban, Dood Arale, and Daroor basins, as well as the Balhaf and Masilah basins in Yemen^12,13,15,16^. The second rift event occurred in the Late Cretaceous, and it was likely caused by a transcurrent motion that resulted in the separation of India and Madagascar^12,13,17^. This event led to the formation of WNW-ESE trending basins, such as the Nogal basin, which are primarily filled with 6–7 km of Upper Cretaceous sediments^15^. The final event took place in the Oligocene and is associated with the separation of Arabia from Africa and the subsequent opening of the Gulf of Aden^12,13,18^. This rift occurred along the Gulf of Aden and reactivated the Guban, Nogal, and Daroor basins. These rift events played a significant role in shaping the crustal architecture of the basins and caused thinning of the crust and upper mantle by up to a factor of two in some basins^12,13,15^.

The tectonic evolution of the sedimentary basins in Somaliland is well explained by a multi-rift extensional model^12,13,15^. As a result, it is anticipated that the crustal structure will show the signature of aborted rift systems and the crust will exhibit a ‘frozen-in’ deformation, which will provide useful insights into the rupture of the continental lithosphere. In this study, we utilized teleseismic records from five seismic stations, along with gravity data acquired across Somaliland, to determine the crustal and upper mantle structure and deformation inherited from the rifts in the region. Receiver function analysis provides an estimate of the Moho depth and *V*_*P*_/*V*_*S*_ ratio. Bayesian inversion of receiver functions offers additional information on the *V*_*S*_ structure beneath the stations. The common conversion point (CCP) imaging and 2D forward gravity modelling along a north–south oriented transect are used to better constrain the crustal architecture in Somaliland. Furthermore, splitting analysis of receiver functions and core-refracted shear waves is employed to explore azimuthal anisotropy within the crust and upper mantle. The findings of this study demonstrate that anisotropy within the crust is controlled by structural inheritance developed during the rift phases, while anisotropy in the upper mantle is generated by large-scale mantle flow. This is the first time, we report the detailed crustal structure, fossil crustal fabrics, and asthenospheric flow beneath the rift basins of Somaliland.

## Results

### Geophysical data

**Figure 1:**
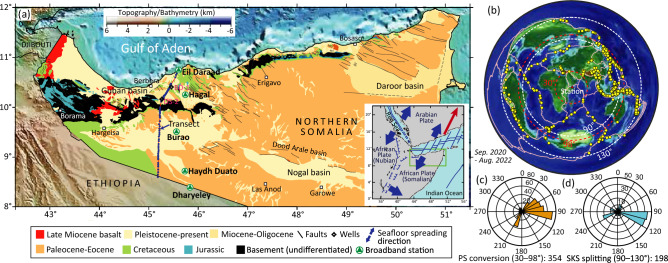
(**a**) Geology map of northern Somalia, modified from^13^. The map shows the location of the five broadband seismometers (green triangles) deployed in Somaliland. SRTM15 + data is used to display the elevation and bathymetry^19^. The location of two drilled wells BD-1 (Biyo Dader-1) and DS-2 (Dagah Shabel-2) are shown. Inset is a location map of Somalia (the green box illustrates the study area) and the surrounding region within the tectonic framework of surrounding plates. The red arrow shows the absolute plate motion of major tectonic plates^20^. (**b**) Azimuthal equidistant projection map centred at the seismic station in Burao showing geographic distribution of teleseismic events (yellow circle). (**c**) and (**d**) Rose diagrams for distance windows of 30–98° and 90–130° showing the number of teleseismic events as a function of backazimuth. Between September 2020 and August 2020, a total of 354 events occurred in the azimuthal distance window of 30–98°, while 198 events occurred in the 90–130° distance window. The figure was generated using Generic Mapping Tools (GMT)^21^.

From December 2019 to August 2022, we collected passive seismic data using broadband seismometers at five stations along a north–south transect in Somaliland (Fig. 1). At each station, a Nanometrics Trillium 120 three-component seismometer with a flat response between 120 s and 150 Hz was deployed at a depth of 1 m. The gravity data were acquired in two separate surveys. The northern part of the gravity data (from Eil Daraad to DS-2 well) was carried out in 2010^11^. The remaining part of the data was acquired in July–August 2021 (Fig. 1). The data were collected using a Scintrex CG5 gravimeter with an instrument reading resolution of ± 0.001 mGal. The distance between the gravity stations in the northern and southern parts was approximately 1 km and 5 km, respectively, but some modification in the sampling distance was needed due to access issues in some places.

### *H-*κ stacking

**Figure 2:**
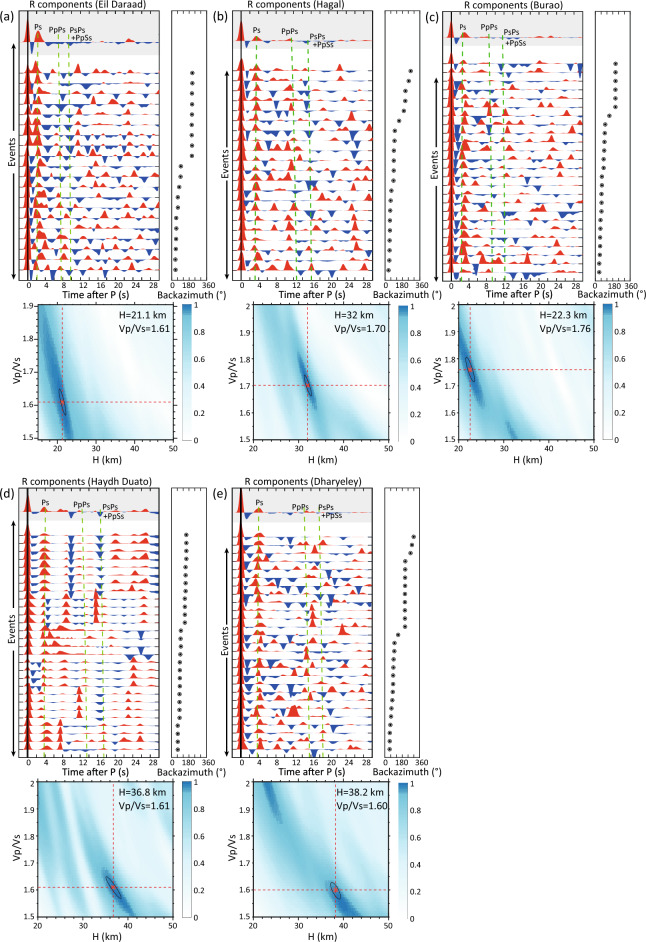
(**a**)–(**e**) Receiver functions for all five stations in Somaliland. The top panels display the receiver functions plotted with increasing backazimuth angle. The stacked receiver functions are shown in grey panels and calculated arrival times for *P*_*S*_ conversion phases (*P*_*S*_*, P*_*P*_*P*_*S*_*, P*_*S*_*P*_*S*_ + *P*_*P*_*S*_*S*_) for the optimal solution in *H-*κ stacking are plotted with green vertical lines. The bottom panels show the Moho depths (*H*) versus *V*_*P*_*/V*_*S*_ ratios (κ) from the *H*-κ stacking technique. The optimum solution for *H* and κ for each station is shown in the bottom panels. Refer to Fig. 1 for the location of the stations.

Receiver function analysis of passive seismic data reveals crustal thickness variation along the north–south transect in Somaliland (Fig. 2 and Table 1). The total number of earthquakes with a moment magnitude (*M*_w_) ≥ 5.5 and epicentral distance of 30–98° that occurred between September 2020 and August 2022 is 354 (Fig. 1b), although the seismic stations only recorded 150 to 260 events. After adopting a signal-to-noise ratio (SNR) $\ge$ 3 and a visual assessment of receiver functions, the backazimuth coverage for these teleseismic events is concentrated between 60° and 90°, and the number of selected radial (R) component receiver functions for *H*-κ stacking per station is 20–30. In the R-component, the coherent *P*_*S*_ phase appears between 2 and 4 s after the *P*-wave arrival (Fig. 2). The multiple reflected waves, *P*_*P*_*P*_*S*_ and *P*_*S*_*P*_*S*_ + *P*_*P*_*S*_*S*_ arrive between 8–13 s and 10–18 s, respectively, and are weaker than *P*_*S*_ phase. The strong positive phases that arrive at approximately 2 s in Eil Daraad and Burao, 3 s in Hagal, and 4 s in Haydh Duato and Dharyeley are the Moho *P*_*S*_, inferring crustal–mantle discontinues (Moho) at depths of 21–38 km and *V*_*P*_*/V*_*S*_ ratios of 1.6–1.76. It is interesting to note that the northern station of Eil Daraad, which is at the shoreline of the Gulf of Aden, reveals a Moho depth of 21.1 km, while Burao station, which is located 150 km inland from the coast, shows an exceptionally shallow Moho depth of 22.3 km with a relatively high *V*_*P*_/*V*_*S*_ of 1.76. This contrasts with the southern stations of Haydh Duato and Dharyeley, which reveal Moho depths of 36.8 km and 38.2 km, respectively. The Hagal station shows a Moho depth of 32.0 km (Fig. 2d and Table 1).

**Table 1:** Moho depth and *V*_*P*_*/V*_*S*_ (κ) for the five stations located in Somaliland.

Station	Latitude (°)	Longitude (°)	Elevation (m)	Moho depth (km)	*V*_P_/*V*_*S*_
Eil Daraad	10.75869	45.60419	12	21.1 ± 1.1	1.61 ± 0.04
Hagal	10.25256	45.74044	382	32 ± 0.9	1.70 ± 0.03
Burao	9.51717	45.56362	1030	22.3 ± 0.5	1.76 ± 0.02
Haydh	8.71734	45.73534	799	36.8 ± 2.0	1.61 ± 0.04
Dharyeley	8.39776	45.83372	762	38.2 ± 1.7	1.60 ± 0.03

### ***V***_***S***_ profiles

**Figure 3:**
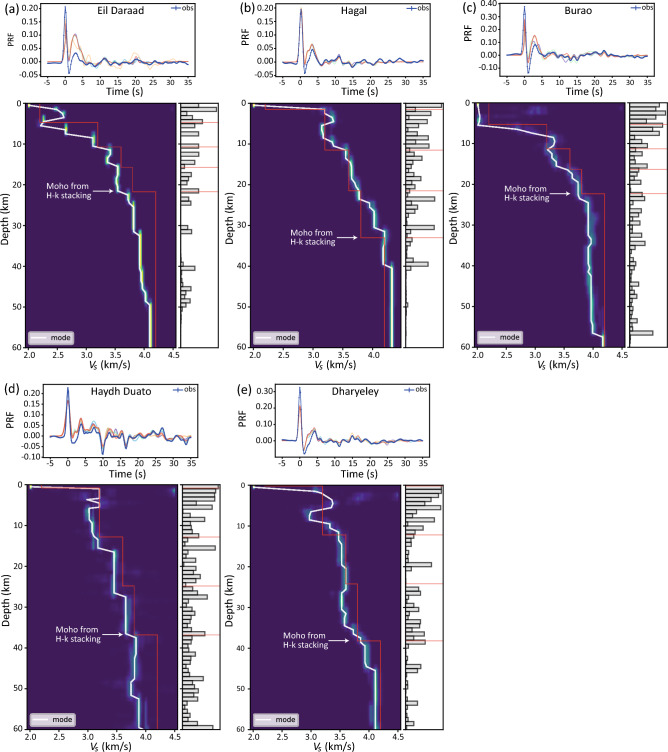
(**a**)–(**e**) *V*_*S*_ structure beneath the five stations obtained from Bayesian inversion of *P*-wave receiver functions. The top panels show the observed (blue curves) and modelled receiver functions. The modelled curves are the ones (out of 21 chains) that converge successfully and retain acceptance rates in the process of multiple independent parameter search paths. The bottom panels show the *V*_*S*_ posterior distributions and corresponding probabilities for interface depth. The red curves are the initial *V*_*S*_ model and the white arrows represent the Moho depth from *H*-κ stacking. A low *V*_*S*_ top layer is noticed under Eil Daraad and Burao stations. At Hagal, Haydh Duato and Dharyeley stations, the *V*_*S*_ values in the top layer match with the upper crustal velocity. The Moho depth from Bayesian inversion matches well with *H*-κ stacking results. Refer to Fig. 1 for the location of the stations.

The *V*_*S*_ profile at station Eil Daraad increases from 2.2 to 2.6 km/s in the top layer down to 6 km depth, and then, after a slight decline, it rises sharply from 2.2 to 3.5 km/s in the 6–12 km depth range (Fig. 3a). The *V*_*S*_ steadily increases in the depth range of 12 to < 32 km with no obvious change in the Moho interface. However, the *V*_*S*_ reaches up to 4.0 km/s at the depth of 32 km, which may indicate that the Moho boundary is relatively deeper compared to the *H*-κ stacking results (Fig. 2a). The *V*s profile for Hagal station, in contrast to that of Eil Daraad station, does not reveal the significant thickness of the top low-velocity layer, but instead shows a gradual increase in velocity from 3.2 to 3.6 km/s in the top 12 km layer and from 3.6 to 4.2 km/s up to 31 km depth (Fig. 3b). The Moho depth, which is slightly shallower than the *H*-κ stacking result, is noted at the interface with a *V*_*S*_ of 4.2 km/s. The presence of 1–2 km of sediments is consistent with drilling data of BD-1 and DS-2 wells, which penetrated the crystalline basement at depths of 1,471 and 1,444 m, respectively. At Burao station, the top 5–6 km layer has a low *V*_*S*_ (2.2 km/s), and at 6 km depth, the velocity suddenly increases from 2.2 to 3.4 km/s (Fig. 3c). The *V*_*S*_ increases from 3.2 to 4.0 km/s, after a slight decrease, up to a depth of 23 km, at which point it remains constant. The Moho depth is marked at the interface with a *V*_*S*_ of 4.0 km/s and matches with the *H*-κ analysis. The low *V*_*S*_ (2.2 km/s) is not seen in the Haydh Duato and Dharyeley stations. The *V*_*S*_ profile at Haydh Duato station remains constant in the upper crust, whereas the lower crust, down to a depth of 36 km, shows a stepwise increase from 3.2 to 4.0 km/s (Fig. 3d). The Moho depth determined from the inversion results agrees well with receiver function analysis. The *V*_*S*_ profile at Dharyeley station shows high and low *V*_*S*_ layers within the upper crust. The *V*_*S*_ is 3.6 km/s between 12 and 34 km depth and increases to 4.0 km/s at 38 km depth. Dharyeley, like Haydh Duato, appears to have a limited (1–2 km) sedimentary cover with low *V*_*S*_ (2.2–2.5 km/s) (Fig. 3e).

### Crustal structure: CCP and gravity modelling

**Figure 4:**
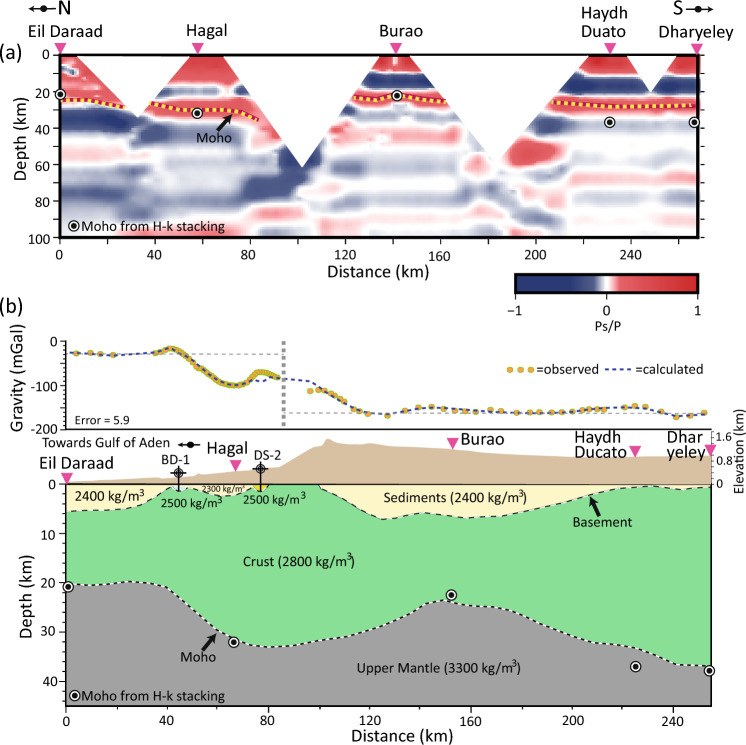
(**a**) CCP imaging along Eil Darrad–Dharyeley transect. Pink inverse triangles show the location of the stations and black-filled circle symbols represent the Moho depths estimated from the *H*-κ stacking. CCP image reveals a major high-velocity gradient between 20 and 30 km depth (yellow dotted line), which is interpreted as the Moho boundary. A good match is noticed for Moho depths observed from *H*-κ stacking and CCP imaging beneath Eil Daraad, Hagal and Burao stations, while shallow Moho depths are seen at Haydh Duato and Dharyeley. Due to wide gaps between stations (white gaps), the shallow intra-crustal discontinuities are unresolved. The scale bar represents the amplitude of positive (red) and negative (blue) polarities of arrivals. (**b**) Two-dimension density model along the transect-A running across Somaliland (see Fig. 1 for location). The Moho depths (black-filled circles) are derived from *H*-κ stacking and densities of sedimentary column constrained are obtained from drilled wells BD-1 and DS-2, which penetrated the Proterozoic basement at 1,471 m and 1,444 m, respectively. The crustal model suggests an asymmetric sedimentary basin in the Burao region with a maximum depth of 6 km. A sauce-shaped basin with a maximum depth of 2 km is noticed beneath Hagal. Beneath Eil Daraad and Burao, the Moho gets shallower (~ 22 km), and the basement is at a 5–6 km depth. The fit between observed and calculated gravity anomaly is particularly good.

The CCP imaging along the profile of seismic stations indicates a high-velocity gradient identified as the Moho boundary, which coincides well with the *H*-κ results (Fig. 4). The crustal thickness increases from 23 km in Eil Daraad to 30 km at Hagal and then reduces to 24 km at Burao (Fig. 4a). The Moho depth is interpreted at 30 km beneath Haydh Duato and Dharyeley stations. This Moho topography is shallower than that determined by the *H*-κ results (37–38 km). At a depth of 10 km, a strong middle-lower crustal interface is found beneath Burao, Haydh Duato, and Dharyeley stations. However, the wide gaps between the stations precluded the resolution of shallow intra-crustal discontinuities (white gap in Fig. 4a).

Two-dimensional forward gravity modelling along the north–south transect delineates the geometry of the crust and rifted sedimentary basins (Fig. 4b). The Bouguer anomaly between Burao and Dharyeley remains relatively constant (− 160 mGal) for 160 km, whereas the gravity anomaly increases to − 30 mGal towards Eil Daraad. The crustal model reveals thick sedimentary basins with densities of 2300–2400 kg/m^3^ beneath Eil Daraad and Burao. The geometry of the basin in Burao seems asymmetrical, with maximum sediment thickness on the northern side. At Hagal station, a saucer-shaped basin with a maximum sedimentary thickness of 2 km is observed. The sediment thickness is thin (< 1 km) at Haydh Duato and Dharyeley stations. The crustal thickness is 36–38 km beneath Haydh Duacato and Dharyeley and it reduces to 24 km at Burao. The crustal thickness increases again to 32 km beneath Hagal and thins to 20 km towards the Gulf of Aden in Eil Daraad.

### Seismic anisotropy

**Figure 5:**
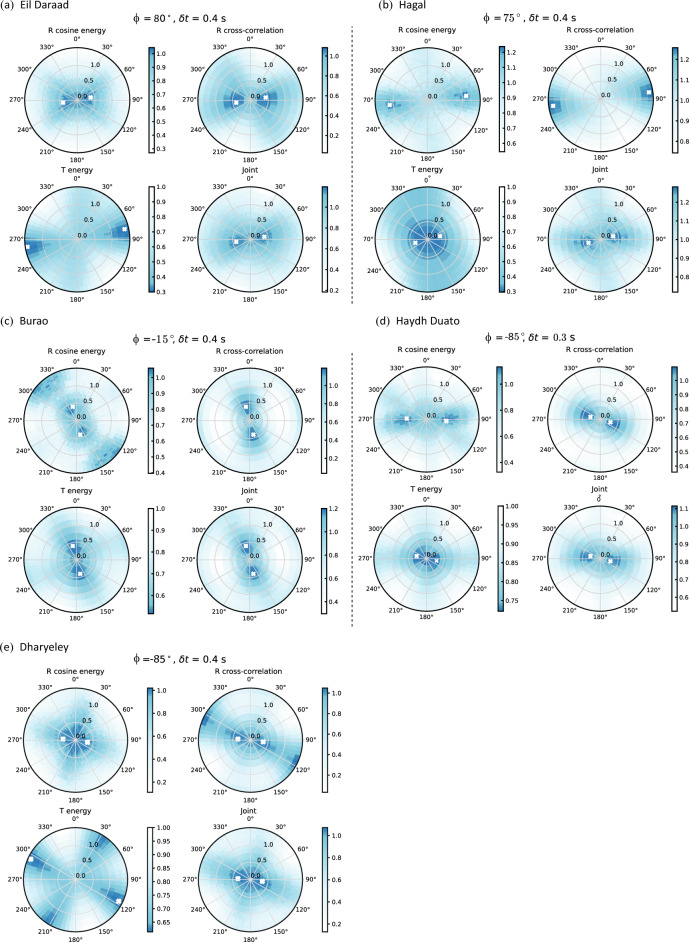
(**a**)–(**e**) Seismic anisotropy results were calculated from three objective functions (R energy maximization with cosine moveout correction, Radial cross-correlation maximization, and T energy minimization) and a joint objective function on R- and T- components of receiver functions. The objective functions are plotted in a 2-D plane of *ϕ* and *δt* in the range of 0–360° and 0–1.5 s, respectively, with an interval of 1° and 0.02 s, respectively. The *ϕ* and *δt* are marked by a white cross symbol. Colour bars indicate variation in objective functions.

A joint analysis technique on the radial (R) and transverse (T) components of the receiver function dataset allowed for determining crustal anisotropy at the five seismic stations. 25–60 *P*-wave receiver functions were selected from among 150–260 events that were recorded based on SNR $\ge$ 3 to offer an accurate assessment of crustal anisotropy. The binned stacked R and T components arranged in ascending backazimuth for each station are shown in Fig. S1. Figure 5 shows three computed individual objective functions and a joint objective function for each station. The anisotropy calculated at Eil Daraad station using R cosine energy maximization, T energy minimization and R cross-correlation maximization shows good agreement with a fast direction (*ϕ*) of 78–80°, (Fig. 5a). The R cosine energy and R cross-correlation techniques estimate a splitting time of (*δt*) 0.4 s, while the T energy method yields a value of 1.3 s. The joint analysis shows *ϕ* of 80° with a *δt* of 0.4 s. At Hagal station, R cosine energy, R cross-correlation, and T energy minimization show good agreement with *ϕ* of 72–83°. (Fig. 5b). The joint analysis yields *ϕ* of 75° and *δt* of 0.4 s. In the case of the Burao station, R cosine energy, R cross-correlation and T energy methods show a good match with *ϕ* of − 22 to − 15° and *δt* of 0.4 s (Fig. 5c). The joint objective function reveals a *ϕ* of − 15° and a *δt* of 0.4 s. For the Haydh Duato and Dharyeley stations, the joint method shows *ϕ* of 95° and a *δt* of 0.3–0.4 s (Fig. 5d,e). However, at Dharyeley station, the T energy minimization deduced a *δt* of 1.3 s. The difference in *δt* deduced from the T energy comparison to R cosine energy and R cross-correlation methods is due to the noise level associated with recorded in these components or the effect of dipping anisotropic layer.

**Figure 6:**
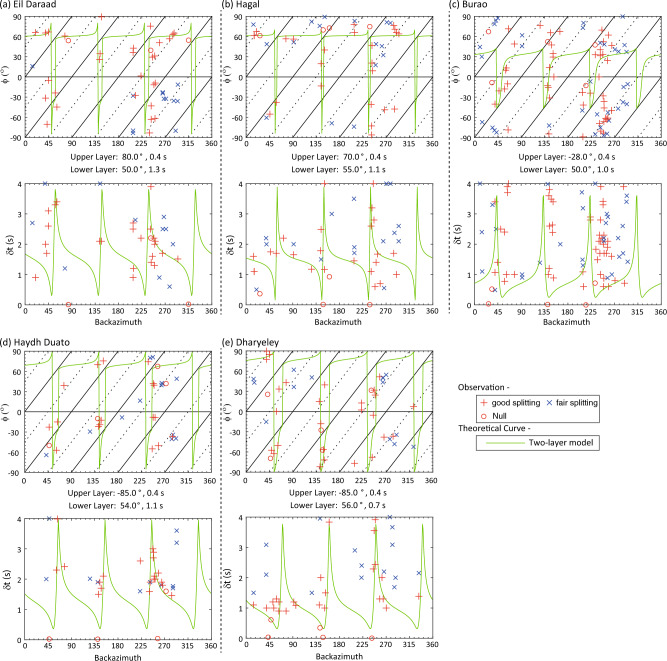
(**a**)–(**e**) SKS splitting analysis results for five stations. Two-layer modelling is performed to fit the observed measurements from SKS splitting. The upper layer anisotropy varies only around the direction of *ϕ* estimated from crustal anisotropy. The top panels show the *ϕ* versus backazimuth and the bottom panels display *δt* versus backazimuth plots. Good and fair splitting measurements are shown with red plus and blue cross symbols, null measurements are shown with red circles, while theoretical curves for the best fit are shown in green colour curves. The parameters of the upper and lower layers from the minimum energy method are placed below the top panel. The direction of *ϕ* ranges from 50 to 56° and delay time is from 0.7 to 1.3 s for the lower layer.

Splitting analysis is also performed on core-refracted shear phases (SKS, SKKS, and PKS, collectively called XKS henceforth) to determine the upper mantle anisotropy from teleseismic data recorded at the five stations. To compute XKS splitting parameters, rotation-correlation and T-component energy minimization approaches were applied on a selected time window to XKS waveforms from teleseismic events that occurred at epicentral distances of 90–130° and had *M*_w_ > 5.7. Around 150–160 seismographs were successfully associated with earthquake events (a total of 198) at individuals linked to seismic stations. The elliptical particle motion is best linearized by the minimum energy method over rotation-correlation (Fig. S2–S4). Therefore, splitting measurements were obtained from the minimum energy method (Table 2). To examine the effect of crustal anisotropy on the XKS splitting measurements, good and fair non-null measurements were fitted with synthetic curves generated by a two-layer anisotropic model (Fig. 6). The splitting parameters (*ϕ*, *δt*) from crustal anisotropy study were used to constrain the upper layer and the *ϕ* and *δt* for the lower layer (i.e., upper mantle) were varied to best fit with measurements. The non-null measurements at Eil Daraad and Hagal stations fit well for an upper layer with a *ϕ* of 70–80° and a *δt* of 0.4s, and a lower layer with a *ϕ* of 50°–55°, and *δt* of 1.1–1.3 s. For the Burao station, the data fit well to an upper layer with a *ϕ* of − 28°and a *δt* of 0.4 s, and a lower layer with a *ϕ* of 50° and a *δt* of 1.0 s. At Haydh Duato and Dharyeley stations, the predicted values of *ϕ* and *δt* for the upper layer are − 85° and 0.4 s, respectively. The splitting parameters (*ϕ* and *δt*) of the lower layer are 54–56° and 0.7–1.1 s. The best-fitting model of SKS splitting data for the five stations has a strong lower anisotropic layer with an average *ϕ* of 50–56°.

**Table 2:** Crustal anisotropy from joint analysis of R and T- components of receiver function and SKS splitting results for the five teleseismic stations in Somaliland.

Station	Crustal anisotropy		SKS splitting			
	Crust		Upper layer		Lower layer	
	*ϕ* (°)	$\delta t$(s)	*ϕ* (°)	$\delta t$(s)	*ϕ* (°)	$\delta t$(s)
Eil Daraad	80 ± 8	0.4 ± 0.10	80	0.4	50	1.3
Hagal	75 ± 10	0.4 ± 0.20	70	0.4	55	1.1
Burao	− 15 ± 6	0.4 ± 0.05	− 28	0.4	50	1.0
Haydh	− 85 ± 8	0.3 ± 0.20	− 85	0.4	54	1.1
Dharyeley	− 85 ± 10	0.4 ± 0.10	− 85	0.4	56	0.7

## Discussion

### Moho topography and ***V***_***P***_/***V***_***S***_ ratio

This is the first study to use receiver function analysis to show lateral variation in the Moho depth across Somaliland (Fig. 2 and Table 1). A thick unstretched crust of 36.8–38.2 km is observed beneath Dharyeley and Haydh Duato in southern Somaliland. The Moho boundary reaches a shallow depth of 22.3 km under Burao, which indicates a decrease towards the north. The Moho depth again deepens and reaches 32.0 km at Hagal and then reduces to 21.1 km at Eil Daraad. The Moho topography variation suggests that the continental crust is stretched more beneath Burao compared to the northern (Hagal) and southern (Haydh Duato and Dheryeley) stations. Moreover, continental extension took place from Hagal (32.0 km) to Eil Daraad (21.1 km), towards the Gulf of Aden. The rapid change in Moho depth is due to the transition of the continental crust beneath the Somaliland margin to the oceanic crust in the Gulf of Aden^12,22^. The results of receiver functions suggest that the unaltered continental crust with a thickness of 37–38 km has been strongly thinned to ~ 21 km to form the rift basins with up to 5–6 km of sedimentary cover. These results agree well with process-oriented gravity and flexure modelling across the conjugate margins of Somaliland and Yemen^12^. Previous receiver function studies from eastern Afar in Ethiopia and Djibouti revealed a crustal thickness of 40 km beneath the African shield e.g.,^23,24^ and Moho depths of 15–25 km along the rift e.g.,^25,26^. Moreover, the inferred crustal thickness beneath the Yemen Plateau is ~ 35 km and it reduces to 22 km near the coast of the Gulf of Aden and 5–10 km within the Gulf of Aden^27^. Additionally, receiver functions and wide-angle seismic data analysis from onshore Yemen, Oman and central Socotra Island reveal an average crustal thickness of 28 –35 km, and crustal thinning towards the Gulf of Aden^28–30^.

The *V*_*P*_*/V*_*S*_ ratios range from 1.6 to 1.76 at the five stations (Fig. 2 and Table 1). The average *V*_*P*_*/V*_*S*_ ratios of the crust beneath the western Yemen Plateau, the northern margin of the eastern Gulf of Aden and Socotra Island are between 1.67 and 1.91^27–29^. The crustal composition in onshore Yemen is felsic with *V*_*P*_*/V*_*S*_ ratios of 1.67–1.77, while the *V*_*P*_/*V*_*S*_ ratio increases up to 1.92 close to the Red Sea coast^27^. The *V*_*P*_*/V*_*S*_ ratio (1.76) at the Burao location is close to the global average for the continental crust (*V*_*P*_*/V*_*S*_ = 1.73;^31^). The high *V*_*P*_/*V*_*S*_ ratio indicates a reduction in the *V*_*S*_ and can be indirect evidence for the presence of fluids or melts within the rift system due to asthenospheric upwelling^26^. The thin crust with a slightly high *V*_*P*_/*V*_*S*_ ratio observed at station Burao implies an enrichment of mafic material. A likely explanation is magmatic underplating/mafic lower crust or the presence of melts generated during the process of rifting. The other possible reason for *V*_*P*_/*V*_*S*_ = 1.76, is the sediment infill in the rifted graben. Furthermore, the inversion of receiver functions reveals a low *V*_*S*_ (2.2–2.6 km/s) at the top layer at Eil Daraad and Burao stations, while the low *V*_*S*_ layer is absent beneath Hagah, Haydh Duato and Dharyeley stations (Fig. 3). Moreover, a sharp change in *V*_*S*_ at the crust–mantle boundary is not observed under Eil Daraad. However, other stations show strong agreement between the Moho depth from the *H*-κ stacking and the inversion of receiver functions. As a result, crustal deformation from the rift phase may have affected the base of the crust and therefore does not result in a notable change in the *V*_*S*_ profile at Eil Daraad.

### Crustal structure across Somaliland

CCP imaging is performed across all the stations to image the Moho and intra-crustal discontinuities (Fig. 4a). The Moho boundary is observed to match very well with the *H*-κ stacking results for Eil Daraad, Hagal and Burao stations. Additionally, a notable intra-crustal discontinuity is observed at a depth of 10 km. The upward trend in the Moho depth is noticed towards the Gulf of Aden and thinned continental crust at Burao. The crustal architecture across Somaliland is also defined using 2D forward gravity modelling (Fig. 4b). The model outlines a 110 km wide sedimentary basin with an average density of 2400 kg/m^3^ and a maximum depth of 6 km in the Burao region. A smaller basin with a density of 2300 kg/m^3^, a width of 40 km, and a maximum sediment thickness of around 1 km, occurs north of Hagal. The Eil Daraad region also has a rift basin with 5–6 km thick sediments that have a density of 2400 kg/m^3^. These findings are consistent with the 3D inversion of gravity and magnetic data in the Dood Arale basin, east of Somaliland, which indicates an NW–SE trending basin with an average density of 2400 kg/m^3,17^. The Dharyeley and Hagal regions appear to have an unaltered continental crust, which has a thickness of 36–38 km, while the Burao and Eil Daraad regions have a thinned continental crust (20–24 km). The stretched continental crust in the interior of northern Somalia is reported in the Guban, Nogal and Daroor basins^11,13,15^. In the Nogal basin, backstripping of biostratigraphic data and process-oriented gravity modelling suggest that the crust thinned down from 35 km at the edge of the basin to 20 km at the basin axis due to the Late Jurassic, Late Cretaceous and Oligocene rift events^15^. Furthermore, the crustal structure derived from backstripping studies of biostratigraphic data and process-oriented gravity and flexure modelling suggests Moho depths of about 30 km at BD-1 and DS-2 wells and thinning of the crust (~ 20 km) along the Somaliland margin of the Gulf of Aden^12^. The thinned continental crust confirms the extension of the continental lithosphere and the rift events that occurred in the region.

### Seismic anisotropy beneath the rift systems

**Figure 7:**
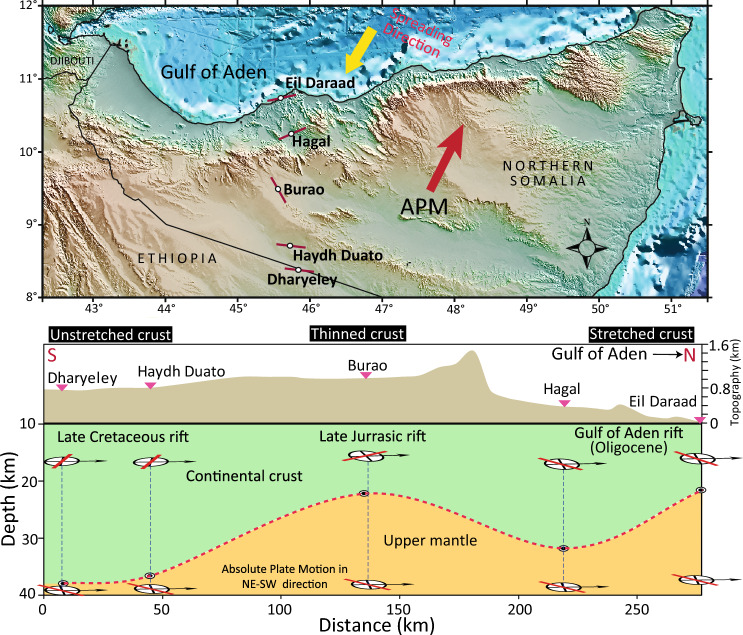
Schematic diagram illustrating the anisotropy observed in the crust and upper mantle beneath the seismic stations. Crustal thickness is from *H*-κ stacking results. The figure was generated using Generic Mapping Tools (GMT)^21^.

Evidence is presented here for the first time for crustal anisotropy produced by rift-related extensional strain and upper mantle anisotropy induced by asthenospheric flow in Somaliland. Table 2 summarizes the results of *P*_*S*_ splitting analysis for the crustal anisotropy and SKS splitting analysis for the upper mantle anisotropy. Figure 7 shows a schematic illustration summarizing the azimuthal anisotropy direction observed in the crust and upper mantle beneath the seismic stations. The stations close to the Gulf of Aden (Eil Daraad and Hagal) display a crustal anisotropy with a fast polarization direction aligned in the rift axis of the Gulf of Aden (Fig. 5a,b). This can be explained by the Oligocene rift event that caused the opening of the Gulf of Aden. The crustal anisotropy in Burao is parallel to the NW–SE trending Late Jurassic rift basins^13^, which suggests a thinned and deformed crust because of the Late Jurassic rift event. Under the unthinned continental crust beneath Haydh Duato and Dharyeley stations (thickness 36–38 km), the crustal anisotropy is observed approximately in the WNW-ESE direction, which is parallel to the Late Cretaceous rift event^15^. This may indicate a preserved far-field response of the Late Cretaceous rift event.

The SKS splitting parameters were inverted for two-layer models, with the upper layer constrained by the parameters of the crustal anisotropy obtained from the *P*_*S*_ splitting analysis of receiver function data (Fig. 6). For the lower layer (i.e., upper mantle), a relatively homogenous pattern in the fast anisotropy directions of 50–56° (NE-SW orientation) is observed with delay times of 0.7–1.3 s. The estimated fast direction of the crust anisotropy in the rift basins of Somaliland is consistent with the observations from other rift systems, such as the main Ethiopian rift, where the splitting direction fast polarization direction is parallel to the rift axis^4,6,25,32^. Laboratory experiments and xenolith studies indicate upper mantle anisotropy is produced by the LPO of olivine crystal resulting from shear strain^8,33,34^. In general, the LPO of olivine crystal develops in response to upper mantle flow or frozen-in lithospheric fabric from past tectonic events^35,36^. The upper mantle anisotropy of all five stations aligns with the regionally occurring NE-oriented direction of the absolute plate motion (APM) of the African and Arabian Plates^20,37^. We believe that the large-scale mantle flow induced by the drag at the base of the lithosphere due to the plate motion plays the main role in producing the upper mantle anisotropy beneath Somaliland. On the other hand, the upper mantle anisotropy directions are also subparallel to the opening direction of the East African Rift, such as the Malawi rift zone^38^. Therefore, it is plausible that the anisotropy of the upper mantle could be due to the superposition of two sub-parallel layers, where the upper layer originates from finite extensional deformation in the lithosphere, and the lower layer is associated with the large-scale mantle flow induced by the APM. The inferences made here suggest that the continental lithosphere of Somaliland has evolved through substantial deformation in the past. A weak lithosphere with elastic thickness, *T*_*e*_, of 5 km is observed in the Nogal basin and Somaliland–Yemen conjugate margins^12,15^. High heat flow at the sub-lithospheric level and substantial internal deformation from multiple rift events may be responsible for the heterogeneous nature of the lithosphere and rheological weakening.

Previous anisotropy studies from East Africa also revealed NE-SW fast polarization directions linked with the LPO induced by plate motion e.g.,^5,6,39,40^. The upper mantle anisotropy of the northern conjugate margin of the Gulf of Aden exhibits NW–SE, NE-SW, and mixed orientations, which were linked to fossil lithospheric mantle anisotropy^41^. However, the upper mantle anisotropy beneath the Arabian Peninsula provided evidence for plate-driven viscous drag in the asthenosphere^42^.

## Conclusions

This study employed a comprehensive array of geophysical techniques, encompassing teleseismic receiver function analysis, Bayesian inversion, common conversion point imaging, and 2D forward gravity modelling, to thoroughly investigate the crustal structure and azimuthal anisotropy along an N-S transect within Somaliland. Our findings reveal a 36.8–38.2 km crust in southern Somaliland, while the central and northern regions exhibit a thinned crust of approximately 21 km overlain by 5–6 km sediments. Joint analysis of receiver functions and shear wave splitting elucidates fast axis directions (50–56°) in the upper mantle, reflecting azimuthal anisotropy aligned with Africa-Arabia plate motion, possibly due to olivine lattice orientation influenced by asthenospheric flow. Furthermore, insights into crustal polarization directions have revealed compelling information. The observed -15° orientation in central Somaliland indicates fossilized deformation linked to the NW–SE trending Late Jurassic rift event. Similarly, seismic stations proximate to the Gulf of Aden exhibit fast polarization directions aligned at 75–80°, indicative of crustal deformation associated with the Oligocene rift event, which played a pivotal role in the inception of the Gulf of Aden's formation. Moreover, the − 85° orientation of crustal anisotropy in southern Somaliland unveils a well-preserved far-field response to the WNW-ESE trending Late Cretaceous rift event. In essence, this study presents insights into rift-induced extensional strain within the crust and asthenospheric flow-induced upper mantle anisotropy. These findings enhance our understanding of rift-related geodynamics and the geological history of Somaliland.

## Methods

### Receiver function analysis

For receiver function analysis, the origin time and location of teleseismic events were downloaded from the United States Geological Survey global earthquake catalogue (Fig. 1b,c). The projected arrival time for the primary *P*-phase is calculated using the TauP module in the ObsPy package^43^. After matching the proper events with seismograms, the raw waveform is cut to 200 s around the projected arrival time. Then, the pre-processing of waveforms is performed by removing the linear trend and average from the raw seismograms before downsampling the data to 25 Hz. Subsequently, the waveform was filtered by applying a Butterworth bandpass filter with 0.05 Hz and 2.0 Hz to remove the high-frequency artefacts. The receiver function analysis is performed in the time-domain deconvolution^44^ and the grid-search algorithm^45^ is used to evaluate the Moho depth (*H*) and *V*_*P*_*/V*_*S*_ (κ) ratio from *P*-wave receiver functions beneath the station. For deconvolution, a 30 s time window is selected and radial components with SNR > 3.0 are retained for *H*-κ stacking. The assumed crustal velocity is 6.5 km/s^46^ and weights to converted *P*_*S*_ phases, *P*_*S*_, *P*_*P*_*P*_*S*_ and *P*_*S*_*P*_*S*_/*P*_*P*_*S*_*S*_ are *w*_1_ = 0.6, *w*_2_ = 0.3, and *w*_3_ = 0.1, respectively. The Moho depth is varied from 20 to 50 km and κ value from 1.5 to 2.0 in the grid-search algorithm. Sensitivity analysis with a distinct set of weights (*w*_1_ = 0.5, *w*_2_ = 0.3 and *w*_3_ = 0.2; and *w*_1_ = 0.7,* w*_2_ = 0.2 and *w*_3_ = 0.1) was performed, and good convergence is observed for each set. The error estimation for the Moho depth and κ is determined from a bootstrap resampling method^47^. The principle of the bootstrap method to quantify the uncertainty is by generating a resampled group randomly, estimating the statistics for resample groups, and 1σ standard deviation is calculated from distribution analysis The receiver function analysis is performed using SplitRFLab toolbox^48^.

A Markov chain Monte Carlo transdimensional Bayesian inversion of *P*-wave receiver functions is adopted to construct a *V*_*S*_ depth profile beneath each station^49^. The initial velocity model consists of the top sedimentary layer, upper, middle, and lower crust and upper mantle with *V*_*S*_ range from 2 to 5 km/s for a vertical depth from the surface of 60 km (Fig. 3). The inversion was performed for a maximum number of layers 20, *V*_*P*_/*V*_*S*_ ratios of 1.6–1.9 and 21 chains, where each chain performing 100,000 burn-in iterations and 50,000 main-iterations with 40–45% acceptance rate. 5% of the complete dataset was rejected as outlier chains, and the final posterior distribution of 100,000 models was assembled based on likelihood. For the inversion of *P*-wave receiver functions, the radial component for the time window − 5 to 35 s is obtained from the stacked receiver function (see Fig. 2) analysis. The present model does not deal with complex crustal structures such as dipping or anisotropic layers at the crustal level.

### Crustal anisotropy and SKS splitting

Splitting analysis of the Moho converted P-to-S (*P*_*S*_) phase on receiver functions is used to investigate seismic anisotropy in the curst^21,50,51^. On the other hand, splitting analysis of XKS phases is used to probe mantle anisotropy^52^. The splitting analysis (of both Ps and XKS phases) assumes that a seismic shear wave splits into fast and slow components with orthogonal polarization directions by travelling through an anisotropic medium. Then, a delay time is accumulated between the two components that are a function of the strength of the anisotropy of the medium and the length of the ray path travelled in the anisotropic layer. In a shear-wave splitting analysis, the polarization direction (*ϕ*) of the fast component and the delay time (*δt*) between the two components are measured as proxies for the direction and strength of anisotropy in the underlying medium.

An integrated technique is used to determine the crustal anisotropy using radial and transverse components of receiver functions. We used the Seispy package to estimate the crustal anisotropy from three individual methods based on radial energy maximization, radial correlation coefficient maximization and transverse energy minimization^21^, and a joint analysis performed on the average of three individual objective functions^53^. In the case of radial energy maximization, the estimation of *ϕ* and *δt* is based on the ratio of peak energy derived by stacking all the radial components of receiver functions after and before the time correction of the cosine moveout in the *Ps* arrival time. The mathematical formula for the ratio of peak energy is presented in^53^. The peak energy ratio is calculated by varying *ϕ* in the range of 0–360° with an interval of 1° and *δt* from 0.0 to 1.5 s with an interval of 0.02 s. The second technique is based on maximizing the radial correlation coefficient for an anisotropy-corrected R-component receiver function. To achieve the anisotropy-corrected components, radial and transverse receiver functions are projected in the assumed fast and slow polarization directions and time shifting (half of the splitting time, $\delta t/2$) is applied in the forward direction for the fast component and in the backward direction for the slow component. The analysis involves a grid search for *ϕ* and $\delta t$ to evaluate the best fit for the crustal anisotropy. The third method, transverse energy minimization, exploits the basic concept of *P*- to *S*-wave conversion at the Moho boundary that reverberation is fully polarized in the radial direction, therefore, the presence of energy in transverse component points to anisotropic nature of the crust^53,54^. For assumed values of *ϕ* and *δt*, the anisotropy-removed transverse receiver functions are stacked and used to evaluate the ratio with the initial T energy. The above-mentioned methods are based on the different properties of the *P*_*S*_ converted phase in response to inhomogeneous structure in the crust and sensitivity to noise present in receiver function data. Thus, a joint analysis is performed to obtain more consistent results of crustal anisotropy from receiver functions^53^. A bootstrap method is implemented to obtain the uncertainty in our estimates.

In XKS splitting analysis, the underlying anisotropic medium is probed using multiple methods such as rotation-correlation^55^, minimum energy, and Eigenvalue technique^52^. The basic principle of all these techniques is to remove the effect of splitting between the two polarized waves in the fast and slow directions by performing a grid search for the parameters *ϕ* and *δt*. We used the Matlab code SplitLab^56^ for data pre-processing and splitting measurements. This code also allows the examination and modelling of the azimuthal variation of the individual splitting parameters for depth-dependent anisotropy beneath seismic stations.

### CCP imaging

CCP stacking technique was carried out along a transect between Eil Daraad and Dharyeley. Receiver functions with SNR > 3.0 were utilized to convert the reverberations phase (*P*_*S*_, *P*_*PS*_, *P*_*SS*_) to depth section following routine procedures: (a) using a 1-D IASP91 velocity model^57^, the receiver functions converted from time (s) to depth (km) and (b) estimation of piercing points of the rays and then projection and stacking amplitudes occurring within a rectangular bin^58^. The *Ps* arrival time and corresponding geographic location are calculated for each receiver function and a linear moveout correction is applied by multiplying the standard slowness factor (± 0.15 s per degree with an increment of 0.01 s per degree) and relative epicentral distance with reference to median epicentral distance. Subsequently, the receiver functions that share common conversion points were stacked based on their epicentral distances. The maximum depth for time-to-depth conversion is considered 800 km with a vertical spacing of 1 km, and a bin spacing interval of 5 km with a width of 100 km.

### Gravity data processing and modelling

The gravity data were corrected for drift, tide, elevation, and latitude. The data were reduced to Bouguer anomaly using an average density of 2670 kg/m^3^. The data was tied to the base station (24.420139° N, 54.502822° E and *g* of 978,883.210 mGal) in Abu Dhabi, United Arab Emirates. The Geosoft^®^ Oasis Montaj software was used to construct a density cross-section along the north–south transect. The model is based on a 2D flat Earth model for gravity computations of structural block and it is extended along the profile to avoid edge effects. We assumed an initial crustal model using results from the *H*-κ stacking, inversion of receiver functions, and CCP imaging. The depth to the basement, thickness and densities of the sedimentary layers are constrained by BD-1 and DS-2 wells. The outcrop geology is utilized to constrain the density cross-section along the transect. The gravity response of the initial model is compared with the observed gravity anomaly and the crustal model was modified until the best fit was achieved between the observed and calculated gravity anomalies.

### Supplementary Information

Supplementary Legends.

Supplementary Figure S1.

Supplementary Figure S2.

Supplementary Figure S3.

Supplementary Figure S4.

## Data Availability

The passive seismic and gravity data were deployed and acquired by the authors. The data are available from the corresponding author.
