# Supplementary material for: Crustal structure and seismic anisotropy of rift basins in Somaliland
Source: Sci Rep. 2023 Oct 14;13:17483. doi: 10.1038/s41598-023-44358-2 (PMC10576820; doi:10.1038/s41598-023-44358-2)
Supplement: Supplementary file 1 — Supplementary Legends. [file 41598_2023_44358_MOESM1_ESM.docx]

**Figure legends**

Figure S1. Binned stacked radial and transverse components of receiver functions for each station arranged by increasing backazimuth angle for each event.

Figure S2. a) and b): Representative diagnostic plots of SKS splitting analysis for a single non-null measurement at Eil Daraad and Hagal stations. The top panels display the seismograms for the initial radial (Q) and transverse (T) components. The central and lower panels display the outcome of the rotation-correlation and minimum energy techniques. The seismogram components in the fast (blue dashed) and slow (red solid) lines, as well as the Q and T components after anisotropy correction, are presented for both methods. Before the correction, the particle motion has an elliptical path, which is linearized after the corrections for anisotropy.

Figure S3. Representative diagnostic plots of SKS splitting analysis for a single non-null measurement at Burao station. The top panels display the seismograms for the initial radial (Q) and transverse (T) components. The central and lower panels display the outcome of the rotation-correlation and minimum energy techniques. The seismogram components in the fast (blue dashed) and slow (red solid) lines, as well as the Q and T components after anisotropy correction, are presented for both methods. Before the correction, the particle motion has an elliptical path, which is linearized after the corrections for anisotropy.

Figure S4. a) and b): Representative diagnostic plots of SKS splitting analysis for a single non-null measurement at Haydh Duato and Dharyeley stations. The top panels display the seismograms for the initial radial (Q) and transverse (T) components. The central and lower panels display the outcome of the rotation-correlation and minimum energy techniques. The seismogram components in the fast (blue dashed) and slow (red solid) lines, as well as the Q and T components after anisotropy correction, are presented for both methods. Before the correction, the particle motion has an elliptical path, which is linearized after the corrections for anisotropy.
