# Supplementary figures and images for: Crustal structure and seismic anisotropy of rift basins in Somaliland
Source: Sci Rep. 2023 Oct 14;13:17483. doi: 10.1038/s41598-023-44358-2 (PMC10576820; doi:10.1038/s41598-023-44358-2)

(a) Eil Daraad

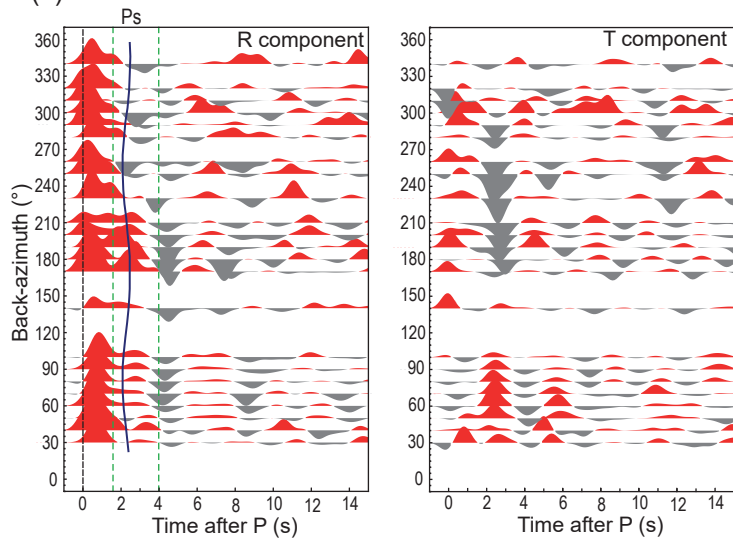

(b) Hagal

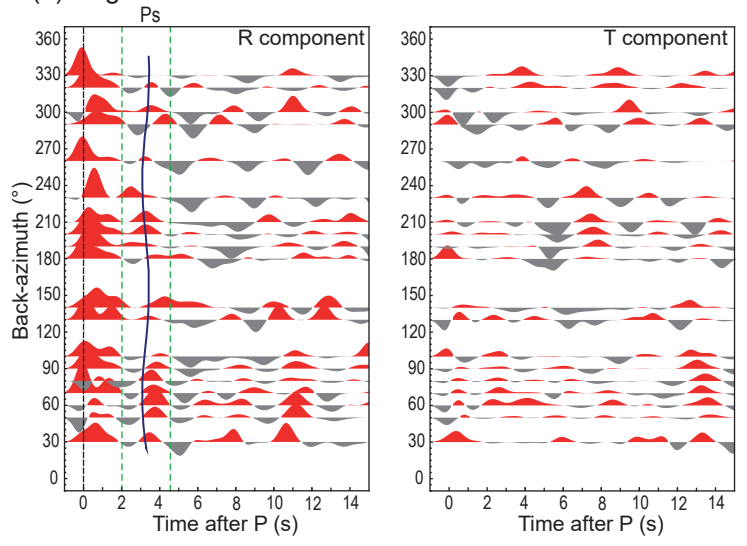

(c) Burao

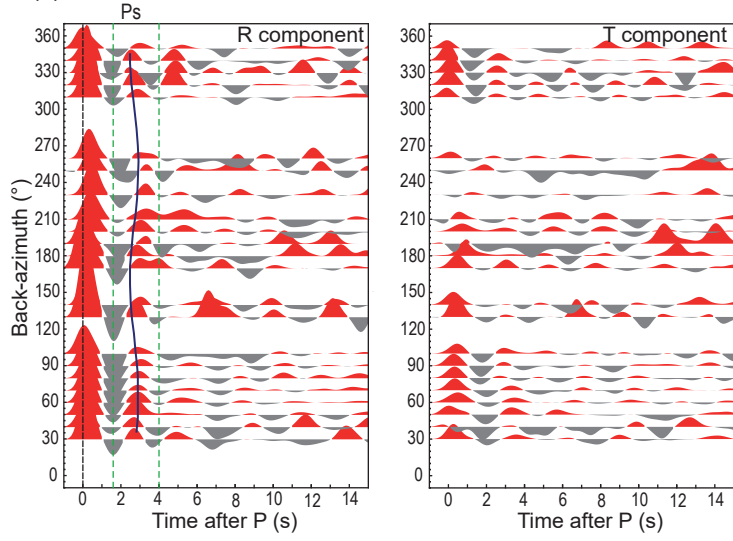

(d) Haydh Duato

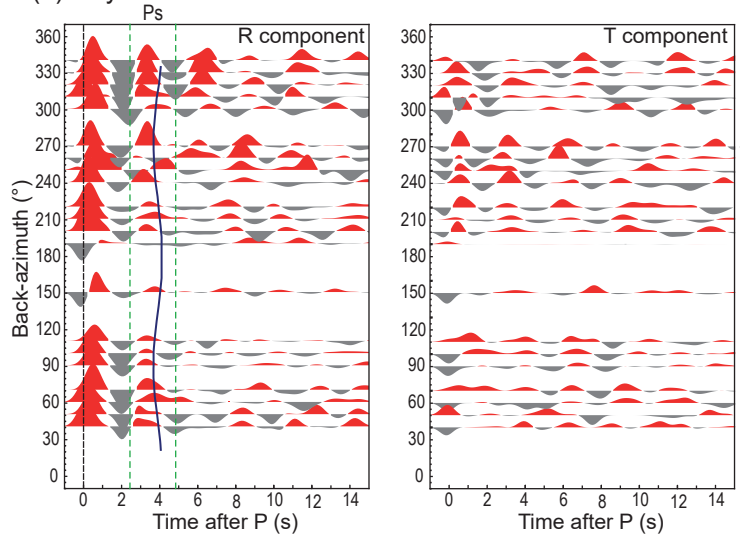

(e) Dharyeley

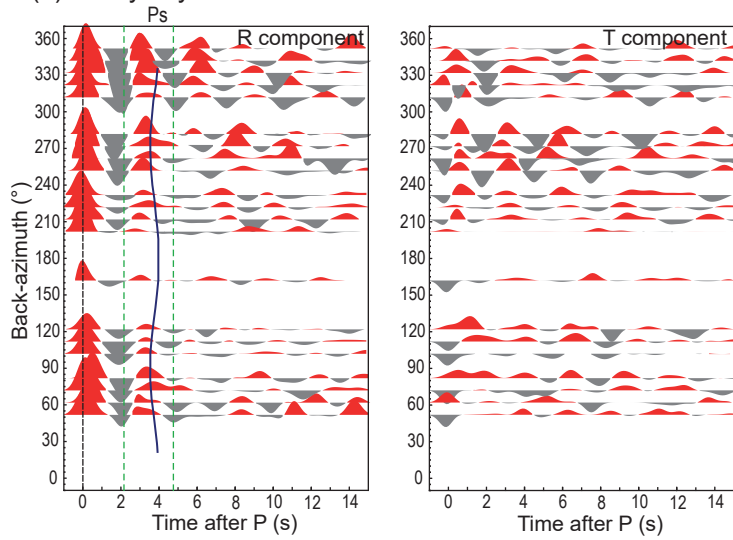

Supplement: Supplementary file 2 — Supplementary Figure S1. [file 41598_2023_44358_MOESM2_ESM.pdf]

a) Eil Daraad

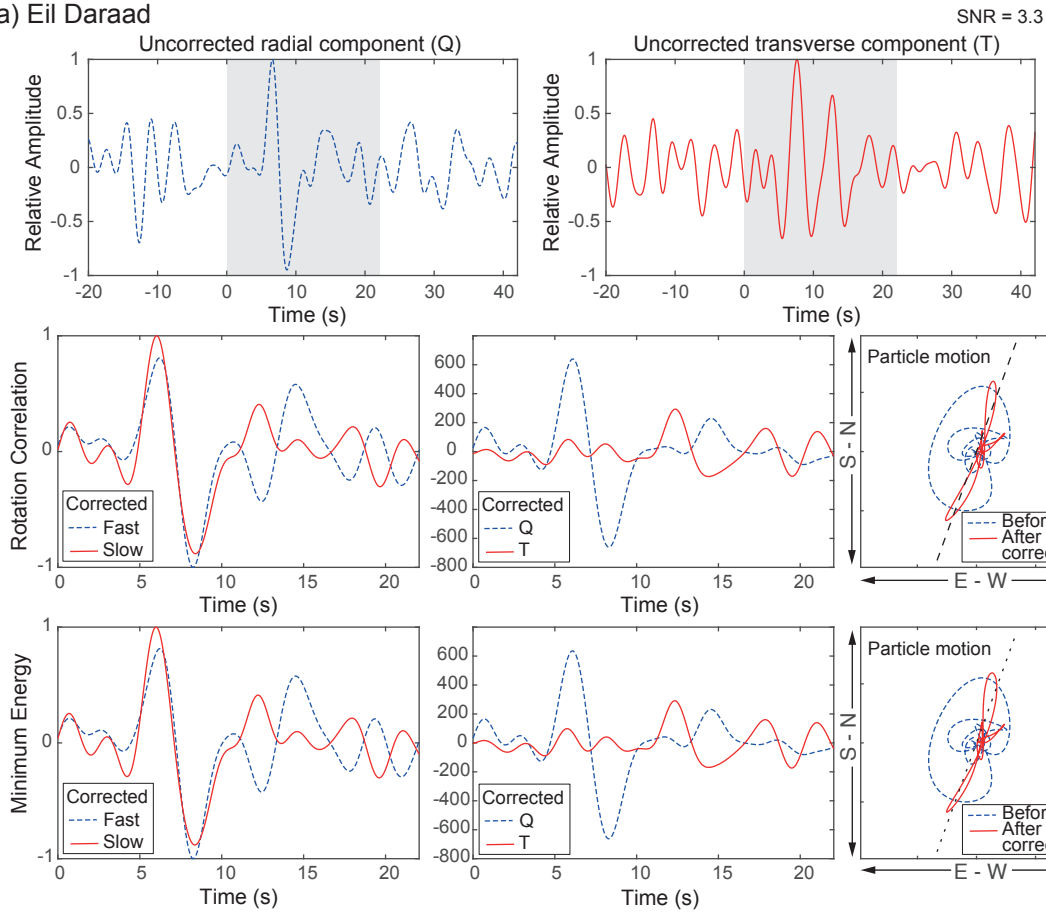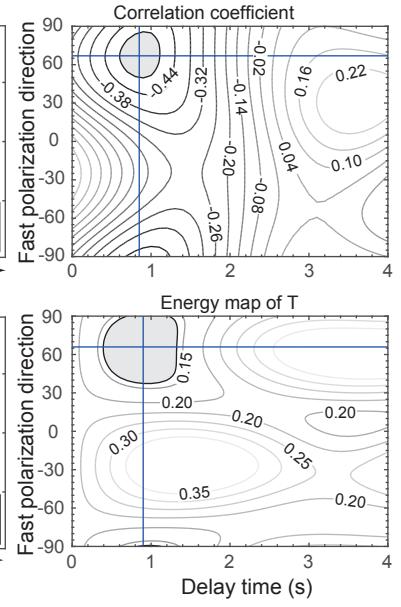

b) Hagal

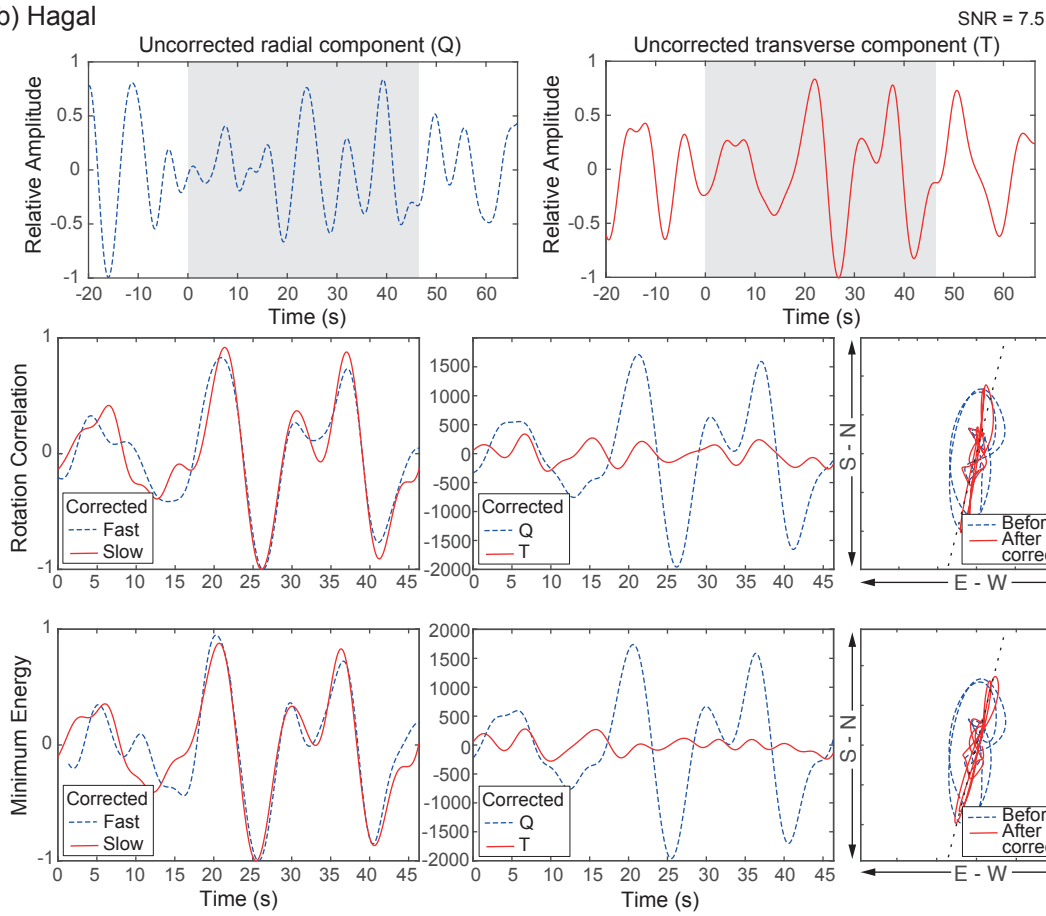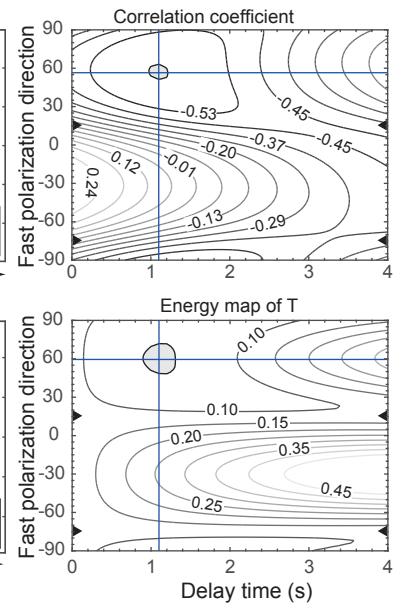

Supplement: Supplementary file 3 — Supplementary Figure S2. [file 41598_2023_44358_MOESM3_ESM.pdf]

Burao

SNR = 9.4

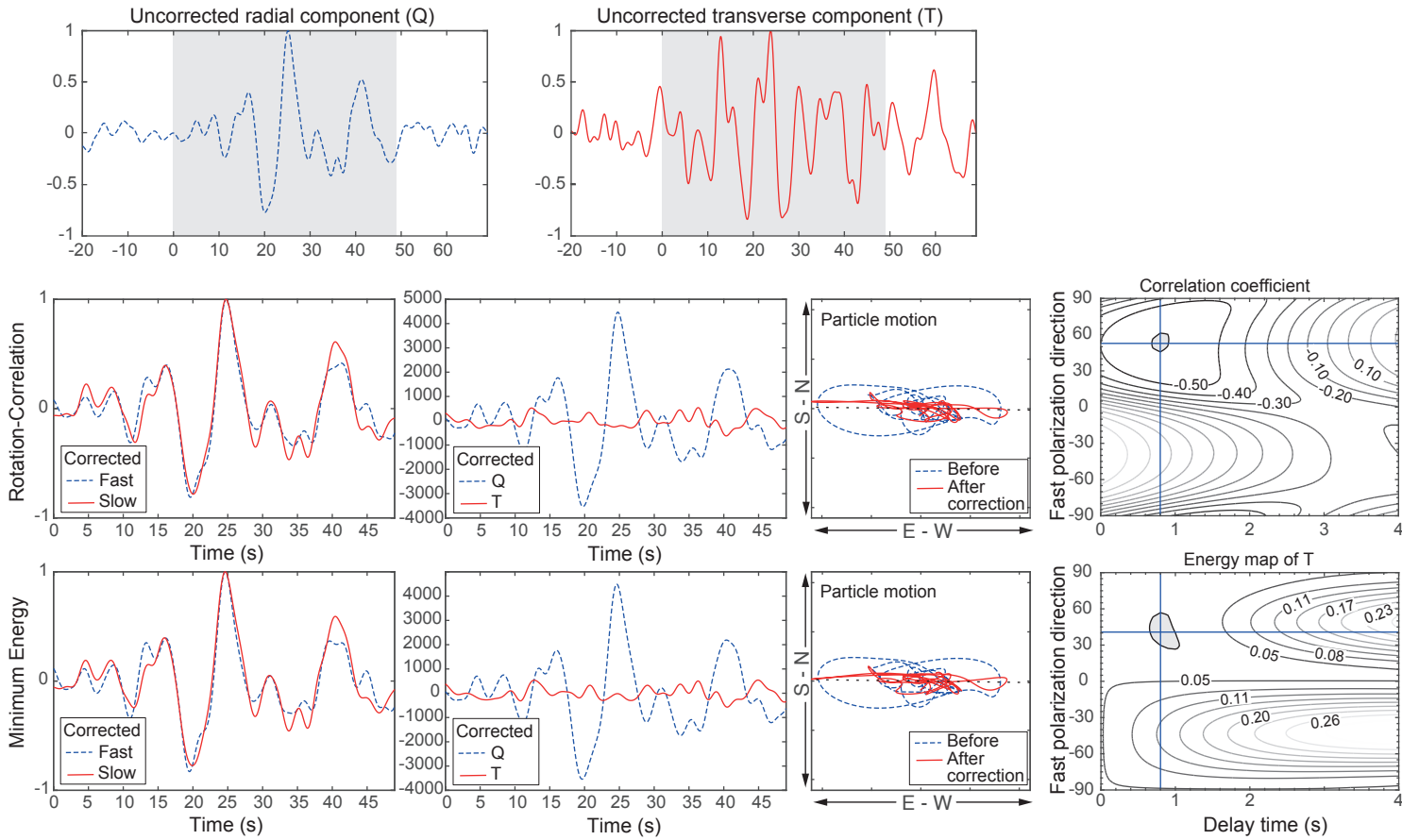

Supplement: Supplementary file 4 — Supplementary Figure S3. [file 41598_2023_44358_MOESM4_ESM.pdf]

### a) Haydh Duato

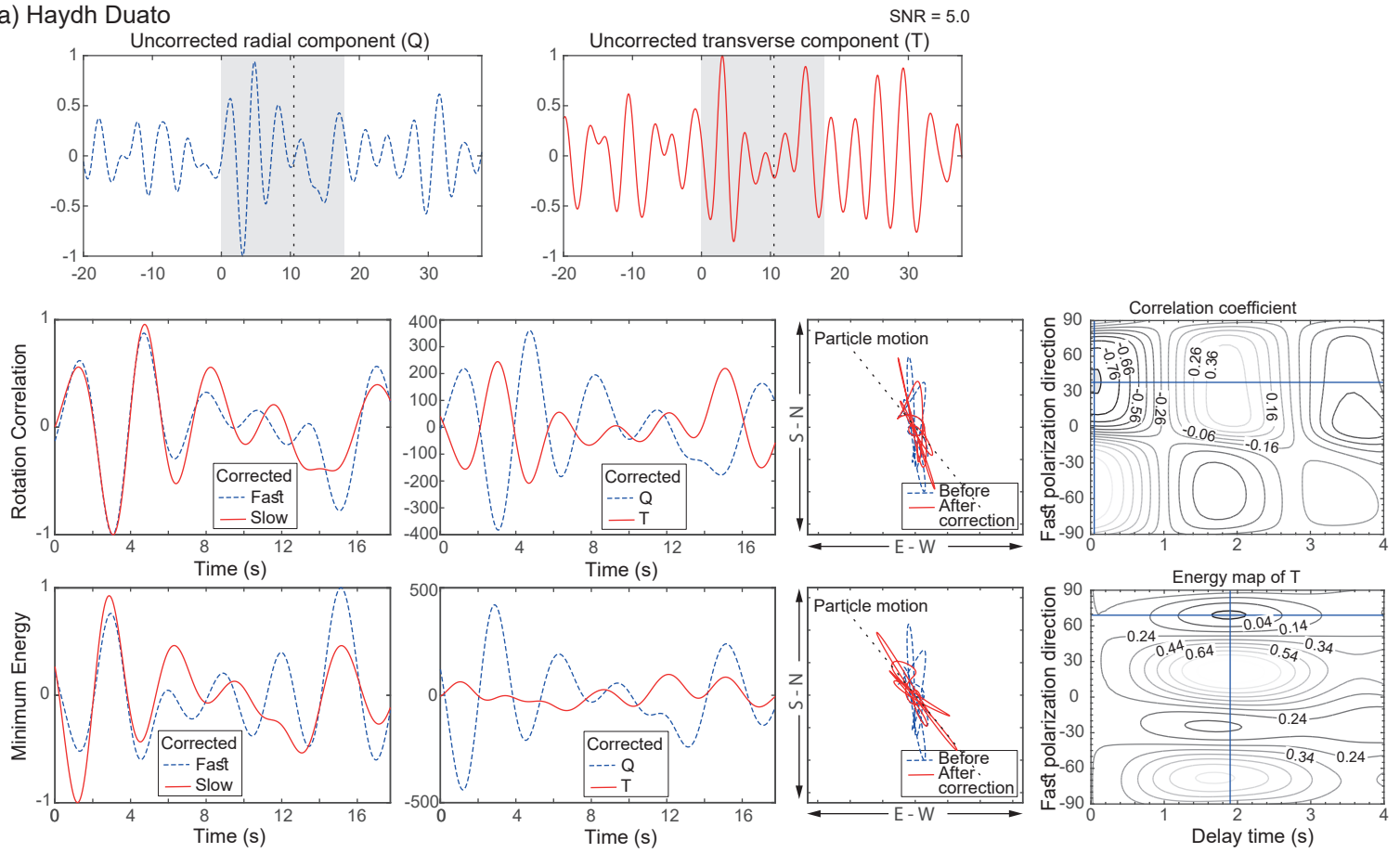

### b) Dharyeley

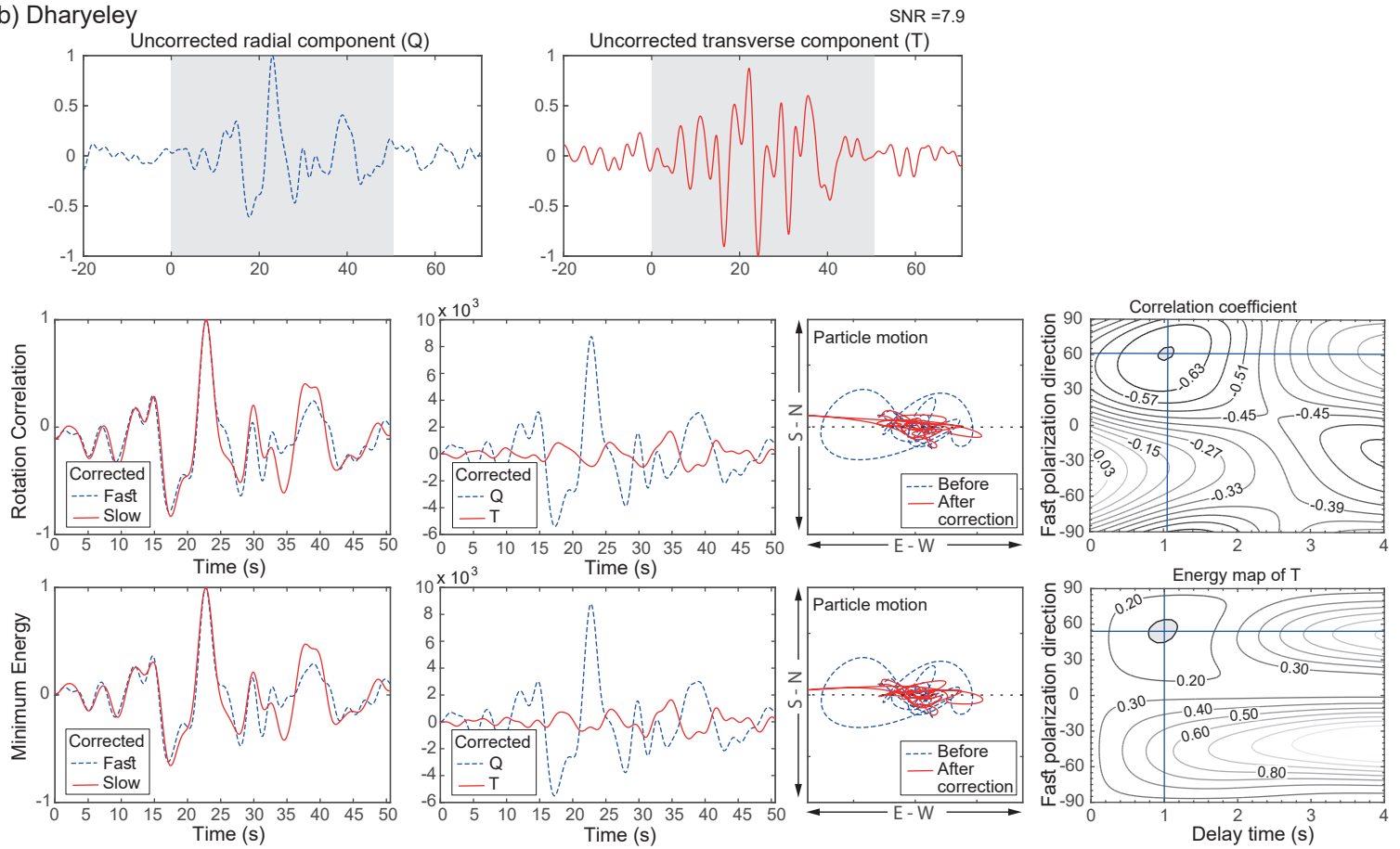

Supplement: Supplementary file 5 — Supplementary Figure S4. [file 41598_2023_44358_MOESM5_ESM.pdf]
